# Facile one-step solvothermal synthesis of active carbon/BiOI microspheres with enhanced visible light-driven photocatalytic activity in the reduction of Cr(vi)

**DOI:** 10.1039/c8ra00169c

**Published:** 2018-02-16

**Authors:** YuanYou Wang, SuoJin Chen, DangQin Jin, AiQin Gong, XueJiao Xu, Changle Wu

**Affiliations:** Department of Chemical Engineering, Yangzhou Polytechnic Institute Yangzhou 225127 China; Testing Center of Yangzhou University Yangzhou 225009 China clwu@yzu.edu.cn 289763548@qq.com +86 0514 87433061 +86 0514 87433061

## Abstract

Active carbon/BiOI microspheres were first prepared using a facile one-step solvothermal route from Bi(NO_3_)_3_·5H_2_O, KI, active carbon, and ethylene glycol. The phase structure, morphology, and optical properties of the as-prepared products were characterized by X-ray diffraction, X-ray photoelectron spectroscopy, high resolution transmission electron microscopy, and UV-visible diffuse reflectance spectra. HRTEM mapping results showed that within the composites, active carbon particles dispersed well onto BiOI spheres. The apparent variations in binding energies and photocurrent measurement results verified that the interactions between both components are strong. As a consequence, these active carbon/BiOI composites exhibit an enhanced photocatalytic reduction activity of Cr(vi) under visible light (*λ* > 420 nm) irradiation when compared with pure BiOI. This work can strengthen the application of BiOI-based micromaterials in treating wastewater contaminated by highly toxic and intractable Cr(vi).

## Abstract 2

Active carbon/BiOI microspheres have promising applications as an efficient visible-light photocatalyst for the treatment of Cr(vi)-polluted water.

## Graphical abstract

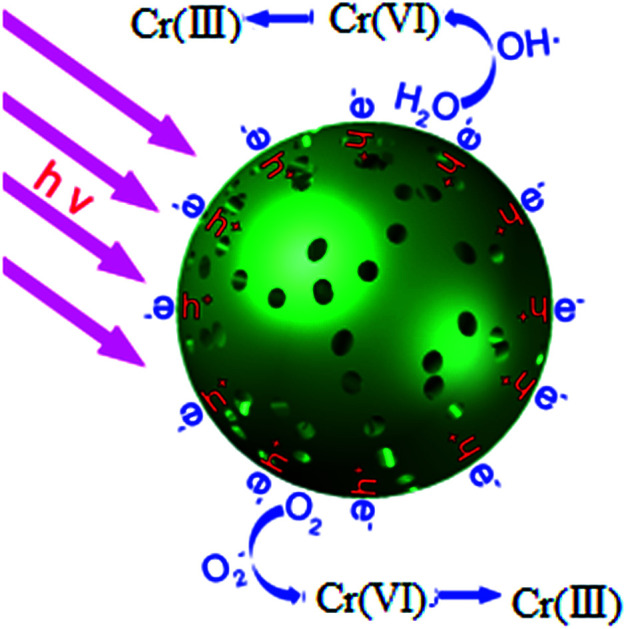

## 1. Introduction

Bismuth oxyiodide (BiOI) as an important narrow band gap semiconductor is usually used in visible-light-driven photocatalysis.^1,2^ However, the fast recombination of photogenerated charges in pure BiOI significantly reduces its photocatalytic activity.^3^ In order to accelerate the separation of photoinduced charge carriers and further enhance the photocatalytic activity of BiOI, many previous studies have focused on the BiOI-based composite photocatalysts, such as BiOI–Bi_2_WO_6_, BiOI–TiO_2_, BiOI–Ag, or BiOI–carbon *etc.*^4–7^ Among them, the BiOI–carbon heterostructure with economic cost and easy preparation was regarded as a promising candidate for solving the above problem.^7,8^ To the best of our knowledge, no reports on carbon/BiOI heterostructure for photocatalytic treatment of Cr(vi), which was a common highly toxic and intractable pollutant in the wastewaters from leather tanning, electroplating, metallurgy, and chromate producing, *etc.* As reported, one-step solvothermal methods have the capability to prepare composite photocatalysts with good mixing, close contact and even strong interaction between different components, which can provide tight and intimate hetero-interfaces for charge transfer and reduce the separation and self-agglomeration of different components during photocatalytic experiment.^9–13^ Herein, we report the synthesis of active carbon/BiOI microspheres by a simple one-step solvothermal route. The carbon/BiOI microspheres has high surface area and strong performance of absorbing Cr(vi).^14^ Furthermore, the photocatalytic activities of the as-prepared pure BiOI and active carbon/BiOI microspheres are also investigated by photocatalytic reduction Cr(vi) in deionised water under the visible light (*λ* > 420 nm) irradiation.

## 2. Experimental

All the chemical reagents used in this work, including Bi(NO_3_)_3_·5H_2_O, KI, active carbon, and ethylene glycol, were of analytical grade. Bi(NO_3_)_3_·5H_2_O, KI and ethylene glycol were bought from Sinopharm Chemical Reagent Co., Ltd. Active carbon powder were bought from Liyang City Liufang Activated Carbon Co., Ltd.

0.04 g active carbon and 0.62 g KI were added into the 80 mL of Bi(NO_3_)_3_·5H_2_O (0.375 mol L^−1^) ethylene glycol solution with continuously magnetic stirring. After stirring 30 min, the mixture was transferred into a Teflon-lined stainless autoclave (100 mL). The autoclaves were sealed and kept at 120 °C for 6 h. After reaction, the precipitates were filtrated, washed with distilled water and ethanol three times, and finally dried in air at 70 °C for 20 h. This sample was called active carbon/BiOI composite. Pure BiOI was prepared without adding active carbon, and other conditions were same with that of active carbon/BiOI composite.

The samples were characterized by XRD (Bruker AXS D8 ADVANCE diffractometer), HRTEM (Holland F-30), N_2_ adsorption/desorption isotherms (Micromeritics Instrument Corporation TriStar II 3020 surface area and porosity analyzer), XPS (Thermo ESCALAB 250Xi), UV-vis absorption spectra (Varian Cary 5000 spectrophotometer), and photocurrent response (German Zahner workstation). Photocatalytic activities of the samples was tested using 0.015 g (300 mL of 50 mg L^−1^) K_2_Cr_2_O_7_ aqueous solution as a probe with the addition of 300 mg of photocatalyst under the irradiation by an 250 W Xe lamp (*λ* > 420 nm). The detailed photocatalytic tests were carried out as in our previous literature.^15^

## 3. Results and discussion

The mole ratio value of active carbon/(active carbon + BiOI) in the as-prepared active carbon/BiOI heterostructure was determined by infrared carbon sulfur analyzer (LECO CS844) to be 48.7 mol%, which was close to the mole ratio of C/BiOI in the reactants.

**Fig. 1:**
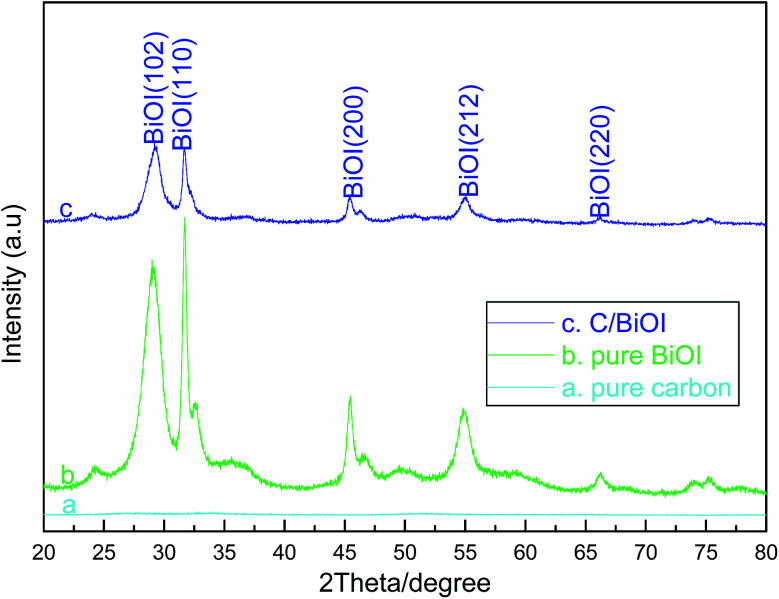
XRD patterns of the as-prepared (a) pure carbon, (b) pure BiOI and (c) active carbon/BiOI.

Fig. 1 shows the XRD patterns of the as-prepared (a) pure active carbon, (b) pure BiOI and (c) active carbon/BiOI composites, respectively. Moreover, the XRD patterns in Fig. 1(b) and (c) display the formation of pure tetragonal phase of BiOI (JCPDS card 073-2062), which indicate that the forming of pure BiOI or BiOI-based composite by this method.

The BET specific surface area of pure BiOI and active carbon/BiOI was 6.92 m^2^ g^−1^ and 16.95 m^2^ g^−1^, respectively.

**Fig. 2:**
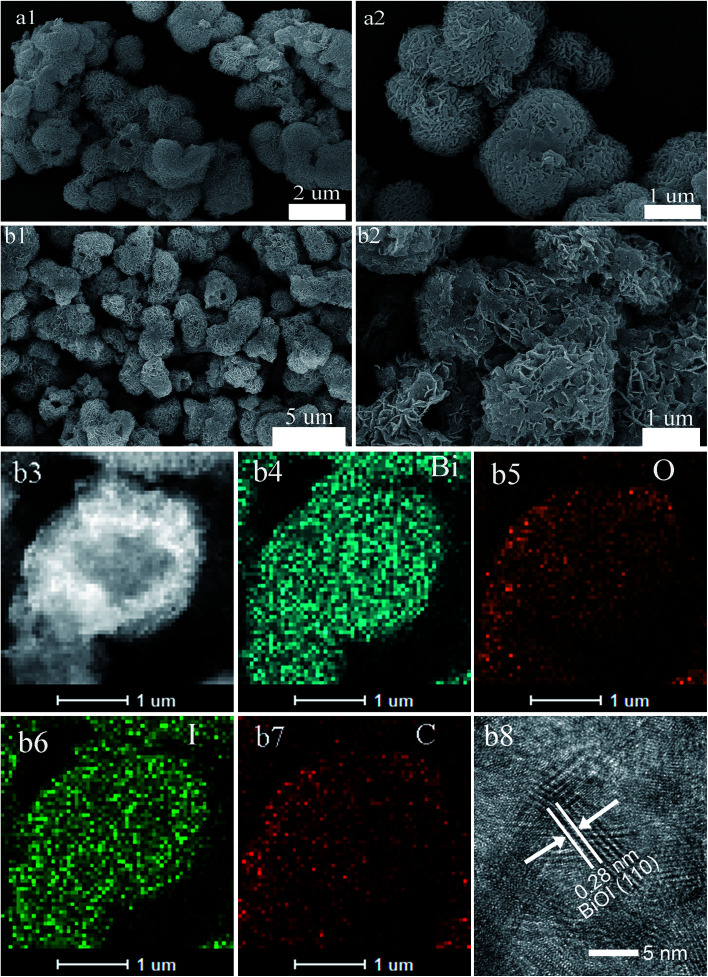
HRTEM images of (a) pure BiOI and (b) the as-prepared active carbon/BiOI microspheres. HRSTEM-EDS mapping results of (b3–b7) the active carbon/BiOI, (b8) HRTEM image of carbon/BiOI microspheres.

Fig. 2(a) and (b) showed HRTEM images of pure BiOI and active carbon/BiOI composite, respectively. Both samples comprised mainly irregular sphere-like particles with particle size 2–5 μm. Besides, HRTEM energy-dispersive X-ray spectroscopy (scanning model of HRTEM, HRSTEM-EDS) mapping results (Fig. 2b3–b7) of the active carbon/BiOI sample indicated that Bi, O, I and C were uniformly distributed on the surface of the sample. Fig. 2b8 shows the HRTEM image of carbon/BiOI microspheres, and the BiOI exhibit good crystallinity. The lattice interval with a distance of 0.28 nm correspond to the (110) lattice planes of the BiOI.

**Fig. 3:**
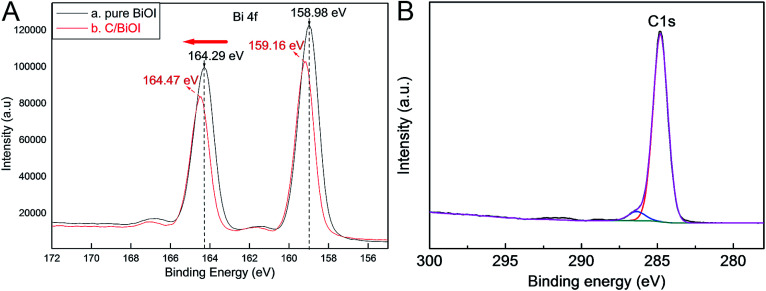
(A) Bi 4f XPS contrast results of the pure BiOI and active carbon/BiOI sample, (B) XPS spectra of C element.

The Bi 4f XPS contrast results of the as-prepared pure BiOI and active carbon/BiOI sample were also studied using a Thermo ESCALAB 250Xi X-ray photoelectron spectroscopy and displayed in Fig. 3A. The apparent variations in binding energies of Bi 4f in XPS results of the samples can be attributed to the strong interactions between two components. This phenomenon can be explained by the fact that the environment of BiOI was altered by active carbon. The variations in binding energies of Bi 4f can also verify the successful forming of active carbon/BiOI heterojunctions by this method. Fig. 3B is the survey spectrum of the C element, wherein the C element can be attributed to the carbon/BiOI microspheres, which is agreement with our experiment. For the XPS spectra of C element, the doublet is located at 284.7 eV, can be ascribed to C1s.

**Fig. 4:**
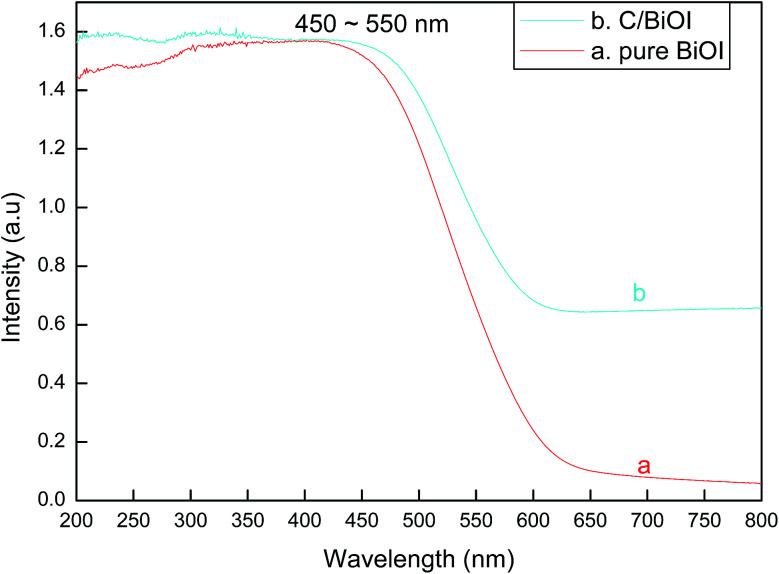
UV-visible diffuse reflectance spectra of (a) pure BiOI and (b) active carbon/BiOI.

From Fig. 4, the band gap edge at the wavelength of about 450–550 nm can be found in the UV-vis diffuse reflectance spectra of both pure BiOI and active carbon/BiOI. When comparing with pure BiOI, it can be found that an enhanced broad tail from about 550 nm to 800 nm appeared in the spectra of the active carbon/BiOI composites. The result implied that the as-prepared active carbon/BiOI composites had strong optical capability almost in the whole range of visible light spectrum. Furthermore, the strong and broad visible-light-response of active carbon/BiOI may indicate that the as-prepared active carbon/BiOI microspheres has the potential to be an efficient visible-light-activated photocatalyst.^16,17^

**Fig. 5:**
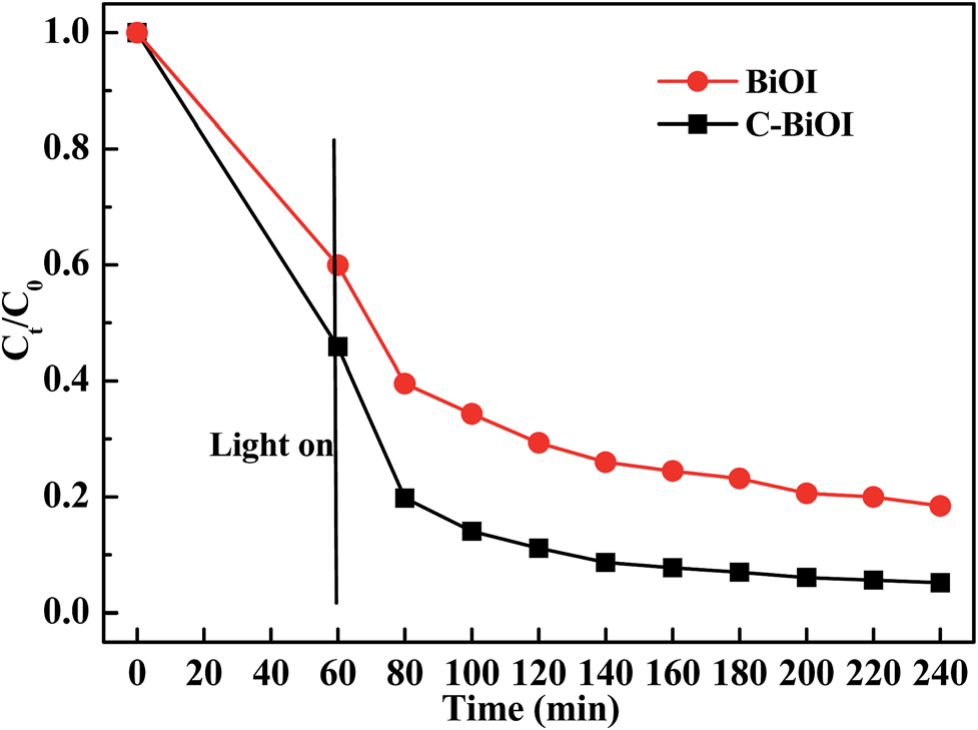
The dark adsorption and visible-light (wavelength longer than 420 nm)-induced photocatalytic reduction of Cr(vi) in aqueous solution using the as-prepared pure BiOI and active carbon/BiOI.

Fig. 5 shows the dark adsorption and visible-light (wavelength longer than 420 nm)-induced photocatalytic reduction of Cr(vi) in aqueous solution by pure BiOI and active carbon/BiOI microspheres. It can be seen from Fig. 4 that all the two products exhibited obvious dark adsorption and visible-light-induced photocatalytic reduction of Cr(vi) in aqueous solution. Active carbon/BiOI microspheres had the larger adsorption of Cr(vi), which may be ascribed to its enlarged specific surface area. Moreover, the photocatalytic activities of the resultant active carbon/BiOI composites was enhanced when comparing with pure BiOI. For example, when irradiated by visible-light (*λ* > 420 nm) for 40 min, the reduction ratio of Cr(vi) in the presence of pure BiOI and active carbon/BiOI were 63.8% and 79.2%, respectively.

**Fig. 6:**
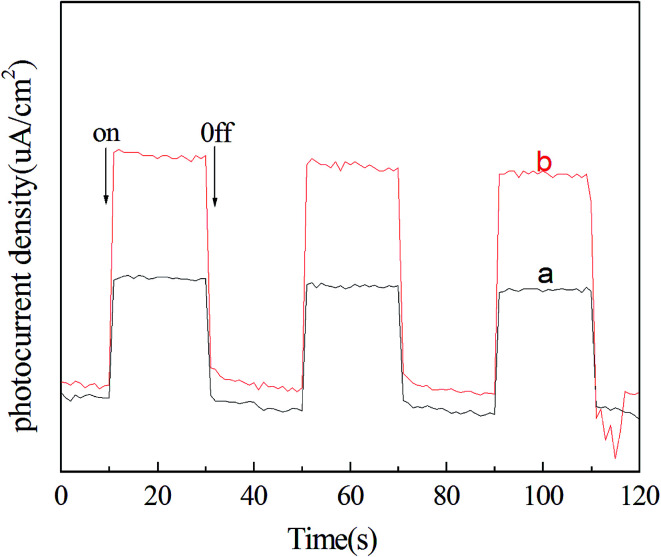
Transient photocurrent response for (a) pure BiOI microspheres and (b) active carbon/BiOI microspheres under visible light irradiation.

To further understand the heterojunction effect on the photocatalytic activity enhancement of the as-prepared pure BiOI and active carbon/BiOI microspheres, I also carefully studied the photo-induced charge transfer properties of the active carbon/BiOI microspheres. Photocurrents for the as-prepared pure BiOI and active carbon/BiOI electrodes were measured on a Zahner workstation (Zahner, German) with a LW405 light (10 mW cm^−2^) as the accessory light source to investigate the electronic interaction between active carbon and BiOI (Fig. 6). It is known that the higher the photocurrent intensity, the more effective the separation of photo-induced carriers.^18^ As shown in Fig. 6, the photocurrent intensity of the active carbon/BiOI microspheres is higher than that of BiOI microspheres, indicating that the active carbon/BiOI microspheres has a more effective separation of photo-induced electrons and holes and faster inter-facial charge transfer, and hence a higher photocatalytic activity. Therefore, the outstanding photocatalytic performance of active carbon/BiOI photocatalyst can be explained by its enhanced surface area, most adsorption of Cr(vi), visible light absorption ability and separation rate of photoexcited charges.

## 4. Conclusions

Active carbon/BiOI microspheres were successfully prepared by a one-step solvothermal process, and confirmed by XRD, XPS, FESEM, BET, DRS, and photocurrent measurement for the first time. The proposed method was facile, mild and cost-effective, which may be suitable for industrial production of active carbon and BiOI microcomposites. The photocatalytic results showed that coupling of active carbon can greatly increase the photocatalytic reduction of Cr(vi) of pure BiOI under visible light (wavelength longer than 420 nm) irradiation. The enhanced photocatalytic performance of active carbon/BiOI microspheres photocatalyst can be due to its enhanced surface area, visible light absorption ability and separation rate of photoexcited charges. The active carbon/BiOI microspheres have promising application as an efficient visible-light photocatalyst for treatment of Cr(vi)-polluted water.

## Conflicts of interest

There are no conflicts to declare.

## Supplementary Material

## References

[cit1] Chen Y. N., Zhu G. Q., Hojamberdiev M., Gao J. Z., Zhu R. L., Wang C. H., Wei X. M., Liu P. (2018). J. Hazard. Mater..

[cit2] Liu Y. B., Zhu G. Q., Gao J. Z., Zhu R. L., Hojamberdiev M., Wang C. H., Wei X. M., Liu P. (2017). Appl. Catal., B.

[cit3] Liu Y. B., Zhu G. Q., Gao J. Z., Hojamberdiev M., Zhu R. L., Wei X. M., Guo Q. M., Liu P. (2017). Appl. Catal., B.

[cit4] Xiang Y., Ju P., Wang Y., Sun Y., Zhang D., Yu J. (2016). Chem. Eng. J..

[cit5] Li J., Zhong J., Si Y., Huang S., Dou L., Li M., Ding J. (2016). Solid State Sci..

[cit6] Wang Y. Y., Gong A. Q., Yu W. H. (2017). Inorg. Chem..

[cit7] Chen Y., Lu Q. J., Yan X. L., Mo Q. H., Chen Y., Liu B. T., Teng L. M., Xiao W., Ge L. S., Wang Q. Y. (2016). Nanoscale Res. Lett..

[cit8] Long B., Huang Y., Li H., Zhao F., Rui Z., Liu Z., Tong Y., Ji H. (2015). Ind. Eng. Chem. Res..

[cit9] Xiao X., Hao R., Liang M., Zuo X., Nan J., Li L., Zhang W. (2012). J. Hazard. Mater..

[cit10] Zou J. P., Luo S. L., Zhang L. Z., Ma J., Lei S. L., Zhang L. S., Luo X. B., Luo Y., Zeng G. S., Au C. T. (2013). Appl. Catal., B.

[cit11] Wang Z., Huang Y., Ho W., Cao J., Shen Z., Lee S. C. (2016). Appl. Catal., B.

[cit12] Yu X., Yang J., Ye K., Fu X., Zhu Y., Zhang Y. (2016). Inorg. Chem. Commun..

[cit13] Wang T., Zhang Y., Ding T. (2014). Mater. Lett..

[cit14] Han J. L., Zhu G. Q., Hojamberdiev M., Peng J. H., Zhang X., Liu Y., Ge B., Liu P. (2015). New J. Chem..

[cit15] Zhang Y., Zhang Q. (2015). Sep. Purif. Technol..

[cit16] Yang S., Xu D., Chen B., Luo B., Yan X., Xiao L., Shi W. (2016). Appl. Surf. Sci..

[cit17] Wang Y. Y., Zhou G. Q., Zhang L., Liu T. Q. (2016). Acta Phys. Sin..

[cit18] Wei H., Zhang Q., Zhang Y., Yang Z., Zhu A., Dionysiou D. D. (2016). Appl. Catal., A.

